# Shift in harms and benefits of cervical cancer screening in the era of HPV screening and vaccination: a modelling study

**DOI:** 10.1111/1471-0528.17190

**Published:** 2022-05-06

**Authors:** Sylvia Kaljouw, Erik E. L. Jansen, Clare A. Aitken, Inge M. C. M. de Kok

**Affiliations:** ^1^ Department of Public Health Erasmus MC, University Medical Center Rotterdam Rotterdam The Netherlands; ^2^ Department of Pathology Erasmus MC, University Medical Center Rotterdam The Netherlands

**Keywords:** cervical cancer screening, HPV screening, modelling study

## Abstract

**Objective:**

To calculate the changes in harms and benefits of cervical cancer screening over the first three screening rounds of the Dutch high‐risk human papillomavirus (hrHPV) screening programme.

**Design:**

Microsimulation study.

**Setting:**

Dutch hrHPV screening programme; women are invited for screening every 5 or 10 years (depending on age and screening history) from age 30 to 65.

**Population:**

Partly vaccinated population of 100 million Dutch women.

**Methods:**

Microsimulation model MISCAN was used to estimate screening effects. Sensitivity analyses were performed on test characteristics and attendance.

**Main outcome measures:**

Harms (screening tests, unnecessary referrals, treatment‐related health problems), benefits (CIN2^+^ diagnoses) and programme efficiency (number needed to screen [NNS]) over the first (period 2017–2021), second (period 2022–2026) and third (period 2027–2031) rounds of hrHPV‐based screening.

**Results:**

The number of screening tests and CIN2^+^ diagnoses decreased from the first to the second round (−25.8% and −23.6%, respectively). In the third screening round, these numbers decreased further, albeit only slightly (−2.7% and −5.3%, respectively). NNS to detect a CIN2^+^ remained constant over the rounds; however, it increased in younger age groups while decreasing in older age groups.

**Conclusion:**

Both harms and benefits of hrHPV screening decreased over the first screening rounds. For younger women, the efficiency would decrease, whereas longer screening intervals would lead to increased efficiency in older women. Programme efficiency overall remained stable, showing the importance of longer intervals for low‐risk women.

**Tweetable abstract:**

Cervical cancer screening: both harms and benefits of hrHPV screening will decrease in the future.

## Tweetable abstract

Cervical cancer screening: both harms and benefits of hrHPV screening will decrease in the future.

## 1 INTRODUCTION

Many high‐income countries have recently made the transition from primary cytology screening to primary high‐risk human papillomavirus (hrHPV) DNA screening in their cervical cancer screening programmes.^1^, ^2^, ^3^ In 2017, the Netherlands became the first country to implement a national cervical cancer screening programme based on primary hrHPV screening for all women, by either clinician‐collected testing or self‐sampling. Women aged 30–60 years are eligible for 5‐yearly invitations. Women who test hrHPV‐negative at age 40 or at age 50 are not invited for screening at age 45 or 55, respectively.

In 2022, the second screening round of the Dutch hrHPV‐based screening programme started, leading to several expected changes in screening effects. First, the hrHPV test is a more sensitive screening test than the previously used cytology test.^4^ This leads to the detection of more high‐grade cervical lesions in the first screening round.^5^, ^6^ For this reason, the prevalence of disease in the eligible population in the second and subsequent screening rounds will be lower than in the first screening round. These effects are commonly seen at the start of a new screening programme, and with the implementation of a more sensitive screening test, we expect to observe similar effects.^7^

Secondly, in the second screening round, the first vaccinated women will enter the Dutch cervical cancer screening programme.^8^ The prevalence of HPV infections and cervical disease in the vaccinated group is expected to be much lower.^9^, ^10^

Lastly, women aged 45 or 55 who tested hrHPV‐negative in the previous screening round will not be invited for screening from 2022. By extending the screening interval for these women, the relative prevalence of disease in the population eligible for screening after the first screening round will increase as lower risk women are removed from the eligible population.

In 2019, a long‐term cost‐effectiveness analysis was published, based on the results from the first year of the Dutch hrHPV‐based screening programme.^11^ However, with the changing screening population, it is important to evaluate the changes in short‐term screening effects per screening round for healthcare planning.

We aim, using microsimulation modelling, to show the cervical cancer screening harms and benefits in the Netherlands during the first, second and third screening rounds of the hrHPV based screening programme. We then compare the effects of the second and third screening rounds with those of the first screening round, to show the relative efficiency of screening in the separate rounds.

## 2 METHODS

To estimate screening effects, we conducted an analysis using the MISCAN‐Cervix microsimulation model. MISCAN‐Cervix is a well‐documented semi‐Markov microsimulation software programme. We used the recently calibrated version of MISCAN‐Cervix described previously by Jansen and colleagues.^11^

### 2.1 MISCAN‐Cervix model

MISCAN‐Cervix generates a large hypothetical population with individual life histories. For this study, we simulated a partly vaccinated population of 100 million women (born 1918–2017) based on Dutch demographic and hysterectomy data.^12^, ^13^ Women in the simulated population can acquire one or more hrHPV infections during their life. These infections are categorised in four groups, based on their oncogenicity and their presence in different vaccine types (i.e. the bi‐, quadri‐ and nonavalent vaccine): (1) HPV‐16, (2) HPV‐18, (3) HPV‐31/33/45/52/58 and (4) HPV‐35/39/51/56/59/66/68. An infection either clears or leads to the development of a pre‐invasive cervical lesion. These lesions can either regress or develop into invasive cervical cancer, classified in International Federation of Gynaecology and Obstetrics (FIGO) stages 1A, 1B, 2, 3 and 4. Multiple infections can occur at the same time, which are independent of each other. Interventions such as hysterectomy, screening and treatment can affect a woman’s life history. Pre‐invasive stages and FIGO 1A cases can only be detected by screening, as these are assumed to be asymptomatic, whereas FIGO 1B or worse can also be clinically diagnosed.

### 2.2 Disease development

The model divides cervical disease into nine sequential stages: hrHPV infection, three pre‐invasive stages (CIN grades 1, 2 and 3) and five invasive stages (FIGO stages 1A, 1B, 2, 3 and 4). The risk of acquiring an hrHPV infection is age‐ and type‐specific. In the model, most HPV infections are transient. Lesions in pre‐invasive stages can also regress. While pre‐invasive lesions can develop without an HPV infection (in which case they will always regress in our model), cervical cancer can only develop in the presence of an hrHPV infection. The durations of HPV infections as well as most pre‐invasive and invasive cancer stages are modelled as exponential distributions with different average durations.

To account for different cancer risk levels for different HPV genotypes, the progression probabilities for the different health stages are dependent on the genotype of the HPV infection (Appendix S1; Table S1). The progression probabilities per group of HPV genotypes are found through calibration.

### 2.3 Screening programme and test characteristics

The hrHPV‐test is the primary screening test in the Dutch cervical cancer screening programme. Women who test positive for hrHPV are then tested for cytological abnormalities (atypical squamous cells of undetermined significance [ASC‐US] or higher). If any cytological abnormalities are found, women are referred to a gynaecologist. HrHPV‐positive women without cytological abnormalities are invited for a repeat cytology test after 6 months.

The test characteristics for cytology were calibrated based on CIN detection rates and interval cancers between 2004 and 2013 (Appendix S1; Table S2). The test characteristics for the HPV test were derived from the literature.^14^, ^15^ The test characteristics for the HPV self‐test were assumed to be equal to those of the regular HPV test. The sensitivity of colposcopy is assumed to be 100%.

### 2.4 Key outcomes

Outcomes of interest can be divided into four categories. The first category is capacity of hrHPV‐testing (self‐tests and GP tests) and cytology testing. Secondly, the benefits of screening are quantified as the number of clinically relevant lesions. We defined clinically relevant lesions as being CIN2 or higher, meaning all referrals resulting in a diagnosis of lower than CIN2 are considered unnecessary. The third category are the harms of screening, quantified as the number of clinically irrelevant referrals, the number of treatments and the number of treatment‐related health problems. We calculated the number of treatments, and the number of treatment‐related health problems based on the results of Aitken et al. and Habbema et al.^16^, ^17^ Lastly, the harms–benefits balance is shown as the number of women needed to screen (NNS) per CIN2^+^ and CIN3^+^ diagnosis, and the number of diagnoses per primary screening test, HPV‐positive screening test, cytology test and referral. All outcomes are presented for the first screening round (2017–2021), second screening round (2022–2026) and third screening round (2027–2031) as the sum over 5 years of hrHPV screening.

### 2.5 Base case analysis

In the base case analysis, we assume attendance rates of primary screening and adherence to repeat testing and colposcopy referral as found in the screening programme (2017 and 2018) (Appendix S1; Table S3).^18^, ^19^ Additionally, we use the microsimulation model STDSIM to find the incidence reductions resulting from vaccination.^20^ The full‐dose vaccination rates reported by the Dutch vaccination programme were used as input (Appendix S1; Table S4).^21^, ^22^

### 2.6 Sensitivity analyses

In univariate sensitivity analyses, we varied several uncertain parameters to investigate their influence on the model outcomes. For screening behaviour, we performed a sensitivity analysis where we applied the attendance rates as observed in 2014–2016 in the Netherlands when a cytology‐based screening strategy was used (Appendix S1; Table S3). In this period, the screening attendance and triage/follow‐up adherence were somewhat higher than in 2017/2018, so in this sensitivity analysis we assume that in the future, the attendance will return to the previous rates again.^18^ In the second sensitivity analysis, we increased sensitivity of the cytology test after a positive HPV test by 50% for CIN1 and CIN2 as compared with the test characteristics in the base case analysis. Higher sensitivity was measured when the cytology test was used as a reflex or repeat test as compared with a primary test.^23^ Thirdly, we adjusted the percentage of self‐tests of all used hrHPV‐tests from 7% to 25%, as a shift to more self‐test attendance is expected as a consequence of the Covid‐19 pandemic. Lastly, we performed a multivariate sensitivity analysis on the sensitivity of the hrHPV self‐test while adjusting the percentage of self‐tests of all used hrHPV‐tests from 7% to 25%. In this sensitivity analysis, we adjusted the test characteristics of the hrHPV self‐test to those found by Inturissi and colleagues, where the relative sensitivity for all lesion grades was found to be lower.^24^

### 2.7 Patient and public involvement

This research was done without patient involvement. Patients were not invited to comment on the study design, interpret the results or contribute to writing or editing of this document. We do not intend to disseminate our results to patients or women eligible for screening.

## 3 RESULTS

### 3.1 Necessary capacity for testing

**FIGURE 1:**
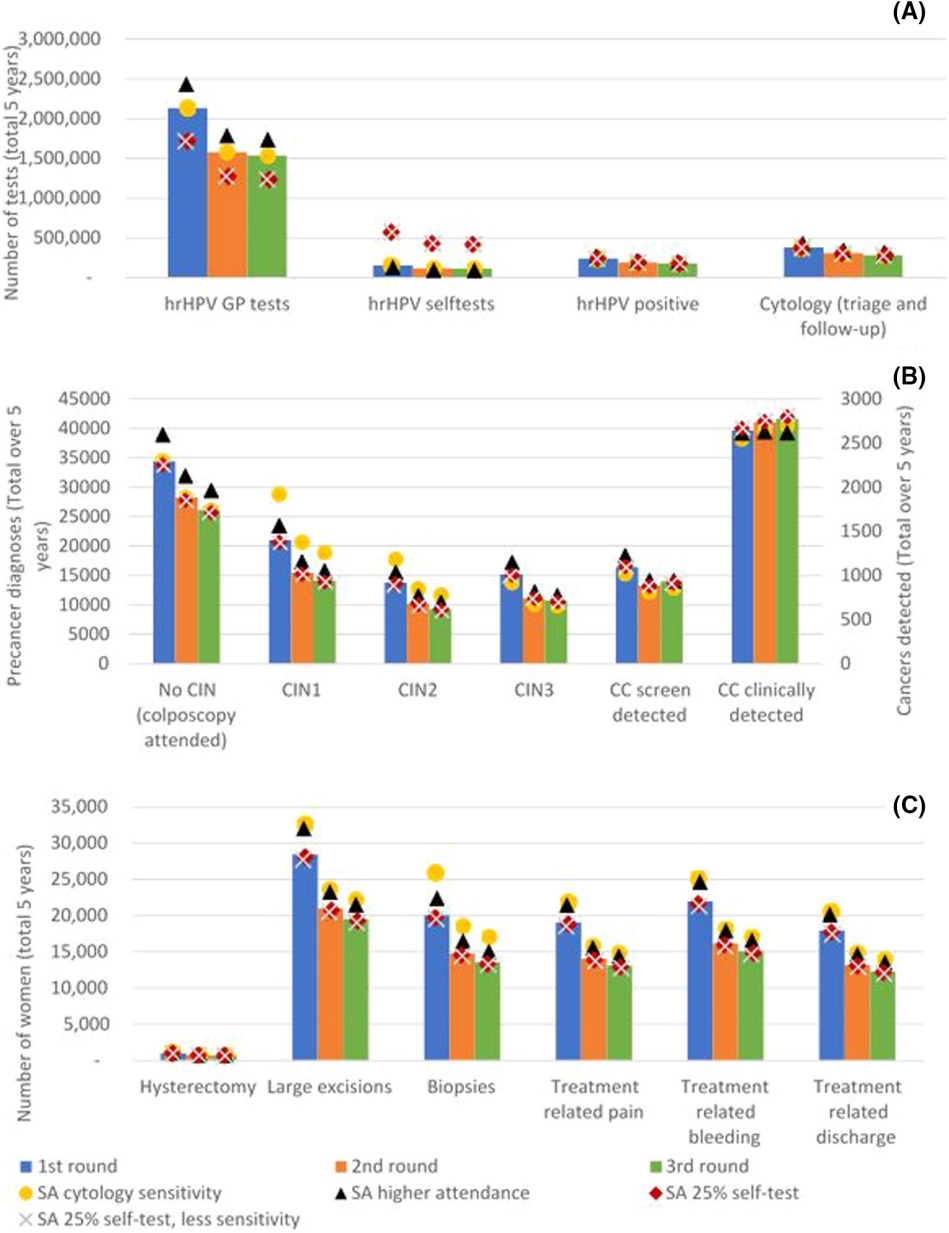
First, second and third screening rounds for the base case analysis and four sensitivity analyses: (A) Number of primary hrHPV (self)tests, hrHPV‐positive tests, cytology tests. (B) Number of pre‐cancer and cancer diagnoses. (C) Number of treatments and treatment‐related health problems. SA cytology sensitivity: Increased sensitivity of cytology test after a positive HPV test by 50% for CIN1 and CIN2. SA higher attendance: Higher attendance rates as observed in 2014–2016 in the Netherlands. SA 25% self‐test: Higher percentage of self‐tests of all used hrHPV‐tests (from 7% to 25%). SA 25% self‐test, less sensitivity: Higher percentage of self‐tests of all used hrHPV‐tests (from 7% to 25%) and lower relative sensitivity of self‐test

The number of primary tests decreases over the first three screening rounds (Figure 1A) for both tests taken at the GP and self‐tests. Especially from the first to the second round, there is a substantial decrease (−26% GP, −22% self‐test), although there is also a small decrease between the second and third screening round (−3% GP, −3% self‐test). These patterns also continue into the numbers of hrHPV‐positive tests and cytology tests, although a slightly larger decrease is visible between the second and third round (−7% and −8%, respectively). The largest decrease in primary tests is visible in the groups of 45–49 and 55–59 year‐olds women (e.g. primary tests taken at the GP: −79% and −85%, respectively [Appendix S1; Table S1b]).

The sensitivity analysis with a higher overall attendance for the primary tests would lead to higher numbers of positive primary tests (+13%) and cytology tests (+13%), but the trend of change between screening rounds remains. A higher assumed sensitivity of the cytology test would not make any substantial difference. When 25% of participating women use the self‐test, this would have an effect on the distribution over primary tests administered by a GP and self‐tests (−20% for GP, +267% for self‐test) but would not have any substantial impact on the number of positive tests or follow‐up tests. In addition, assuming a lower sensitivity for the self‐test would not make a considerable difference when 25% of women use the self‐test.

### 3.2 Benefits of screening across the screening rounds

The number of CIN2/3 lesions found decreases substantially after the first round (−27% for CIN2, −26% for CIN3), followed by a small decrease after the second round (−9% for CIN2, −4% for CIN3) (Figure 1B). The number of screen‐detected cancers decreases substantially after the first screening round (−19%) but then increases by 5% after the second screening round. The number of clinically detected cancers increases by 4% after the first screening round and another small increase (+1%) is observed after the second round. The largest decrease in CIN2^+^ diagnoses is visible in the youngest age groups (30–34: −39% and 35–39: –29% [Appendix S2; Table S1b]). On the other hand, we observed an increase in CIN2^+^ diagnoses in the oldest age group (60–64: +25% [Appendix S2; Table S1b]).

When a higher attendance for primary screening was assumed, it led to an increase in the number of diagnoses for CIN2 and CIN3 (+11%). Higher attendance would lead to a similar increase in the number of screen‐detected cancers (+6%), whereas the number of clinically detected cancers decreases (−4%). An increase in cytology sensitivity for CIN1 and CIN2 would cause a large increase in number of CIN2 lesions found (+27%). As these lesions are detected earlier, this leads to a decrease in the number of detected CIN3 lesions (−9%) and screen‐detected and clinically detected cancers (−8% and −3%). Assuming that 25% of participating women use the self‐test, with or without the assumption of lower relative sensitivity of the self‐test, does not have a considerable impact on the number of detected CIN2^+^ lesions.

### 3.3 Harms of screening across the screening rounds

The number of clinically irrelevant lesions (no CIN, CIN1) also decreases substantially after the first screening round (no CIN: −18%, CIN1: −27%), followed by a smaller decrease after the second screening round (no CIN: −7%, CIN1: −9%) (Figure 1B). In the sensitivity analyses we found that higher attendance for primary screening would increase the number of clinically irrelevant referrals (no CIN: +13%, CIN1: +12%). An increase in cytology sensitivity for CIN1 and CIN2 would cause a large increase in number of CIN1 lesions found (+35%).

The number of hysterectomies, large excisions and biopsies also decreases after the first screening round (−26% for all), followed by a small decrease after the second screening round (−6%, −7% and − 8%, respectively), due to the decrease in referrals and diagnosed CIN lesions (Figure 1C). As a result, treatment‐related pain, bleeding and discharge would also decrease in future screening rounds (−31%). A higher attendance rate would result in more treatments and treatment‐related issues over all screening rounds compared with the current attendance rates (+11%). Higher cytology sensitivity for CIN1 and CIN2 would also result in a higher number of treatments, especially the number of biopsies (+27%). The effects on the number of treatment‐related health issues are slightly larger than those of higher attendance (+14%). The assumption of 25% of participating women using the self‐test, with or without the assumption of lower relative sensitivity of the self‐test, does not have a considerable impact on the number of treatments and treatment‐related health issues.

### 3.4 Harms–benefits balance of screening across the screening rounds

**FIGURE 2:**
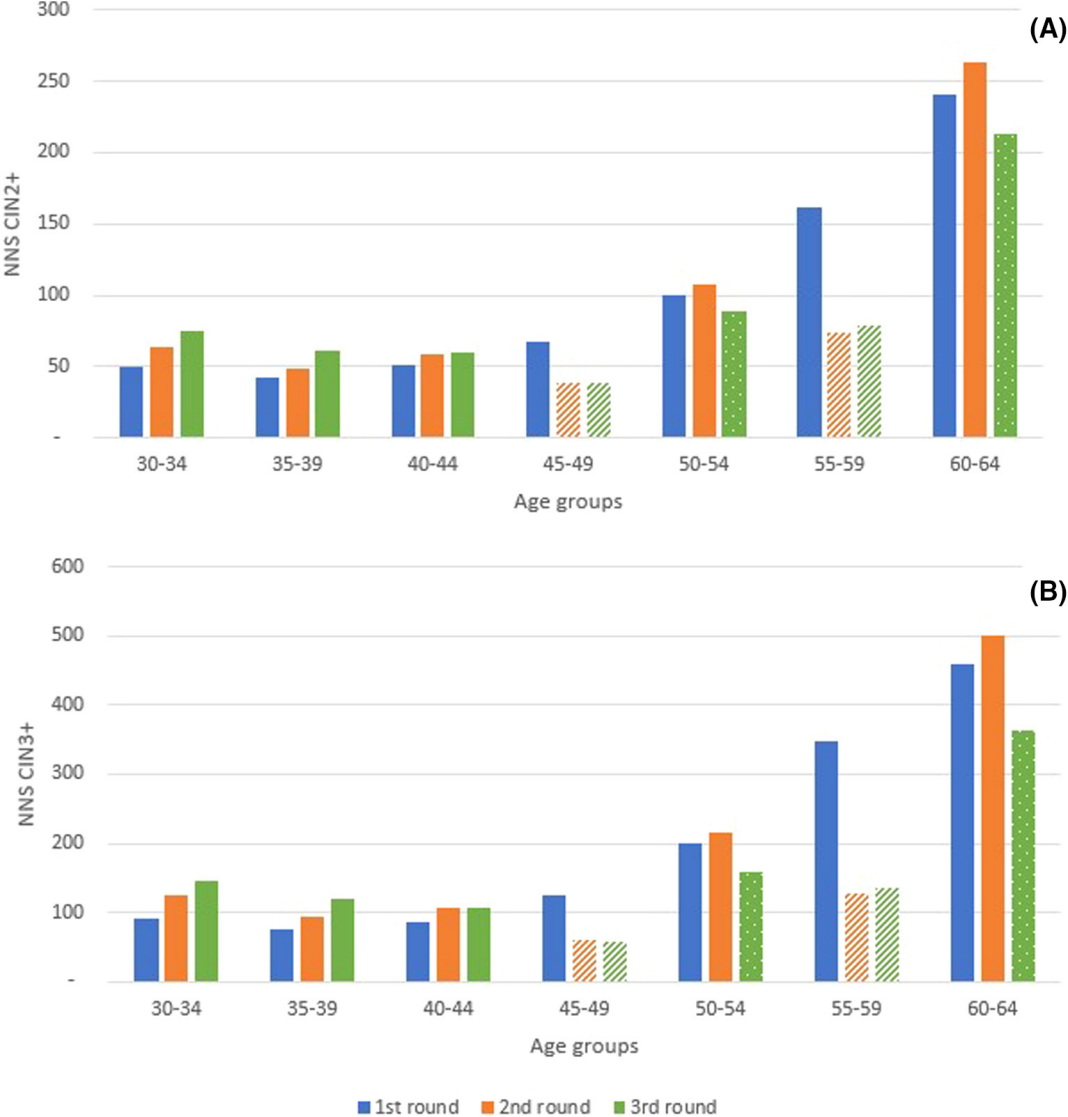
Number needed to screen (NNS) to detect one CIN2^+^ diagnosis (A) or one CIN3^+^ diagnosis (B) by age group. Screening age groups with a higher expected disease risk are marked with either arching (i.e. only invited if HPV positive in the previous round) or dotting (i.e. first women screened after a 10‐year interval)

The efficiency of the screening programme as illustrated by diagnoses per hrHPV‐test, positive HPV‐test, cytology test and referral show a decreasing trend, although there is some fluctuation visible over the three rounds (Table 1). Accordingly, NNS for one CIN2^+^ diagnosis and NNS for one CIN3^+^ diagnosis both show a slightly increasing trend. In the results per age group, we see that there is an increase in the NNS CIN2/3^+^ over the rounds for the youngest age groups (30–39), whereas there are more fluctuations in this measure of efficiency in the higher age groups (45–64) (Figure 2A,B; exact numbers in Appendix S2; Tables S2a,b). Specifically, in age groups 45–49 and 55–59, the NNS of CIN2^+^ and CIN3^+^ decrease after the first screening round. In age groups 50–54 and 60–64, the NNS increases after the first screening round, whereas it decreases in the third screening round for both CIN2^+^ and CIN3^+^.

**TABLE 1:** Efficiency of the programme as shown by the CIN2^+^ diagnoses per hrHPV test, hrHPV‐positive test, cytology test and referral

	Efficiency of the programme			Difference compared with 1st round (%)	
	1st round	2nd round	3rd round	2nd round	3rd round
CIN2^+^ diagnoses per hrHPV test	0.013	0.013	0.013	−0.3%	−3.8%
CIN2^+^ diagnoses per hrHPV positive	0.126	0.118	0.119	−6.4%	−5.8%
CIN2^+^ diagnoses per cytology test	0.079	0.073	0.074	−8.0%	−6.4%
CIN2^+^ diagnoses per referral (PPV)	0.350	0.338	0.342	−3.5%	−2.3%
NNS CIN2^+^	76	76	79	0.3%	3.9%
NNS CIN3^+^	140	141	142	0.6%	1.2%

*Note:* Additionally we show the efficiency of the programme with the number needed to screen for a CIN2^+^ diagnosis and per CIN3^+^ diagnosis. The right‐hand side of the table shows the percentage difference compared with the first screening round.

Abbreviations: NNS, number of women needed to screen; PPV, positive predictive value of a referral.

## 4 DISCUSSION

### 4.1 Main findings

The aim of this study was to give an insight into the expected change in harms and benefits over the first three rounds of the Dutch hrHPV‐based screening programme to aid in informing long‐term health services planning. We found that the number of primary tests decreases by 26% after the first screening round, and the number of CIN2^+^ diagnoses decreases by 24%. In the third screening round, the number of primary tests is 3% lower than in the second screening round, whereas the difference in number of CIN2^+^ diagnoses is −5%. As a result, the number of treatment‐related health problems decreases by 31% over the first three screening rounds. As the decrease in the number of primary tests and the number of CIN2^+^ diagnoses are similar, the overall NNS CIN2^+^ remains stable. However, results per age group show that the NNS of CIN2^+^ increases in younger age groups, whereas it decreases in older age groups.

The decrease in primary screens, diagnoses and treatment‐related health problems is caused by three main factors. The foremost factor is the switch from a 5‐year interval to a 10‐year interval for HPV‐negative women at 40 and 50 years of age. This is the main reason why the balance between harms and benefits remains the same over the first three screening rounds. However, this is also the cause of an increase in interval cancers, as shown by an increase of 5% in clinically detected cancers over the first three screening rounds. Secondly, as the Dutch cervical cancer screening programme switch to a more sensitive screening test (from primary cytology screening to primary hrHPV‐screening), many more prevalent CIN lesions are being detected and removed from the population, similar to starting a new screening programme.^7^ This leaves mainly incidental lesions after the first rounds, as shown clearly by the increase in NNS to detect a CIN3^+^ in age groups <45 years old. Thirdly, we observed a small effect of vaccination starting in the second screening round. This effect is mainly visible in the decrease in the number of CIN2^+^ lesions in the youngest age group (30–34 year‐olds: −38.7% over the second and third screening rounds).

Surprisingly, none of the expected changes in harms and benefits in the near future show any remarkably change in the harms–benefits ratio (in terms of NNS) of cervical cancer screening. However, the efficiency of screening will change per age group; for younger age groups (≤44 years old) the NNS increases under the influence of vaccination (the oldest vaccinated women are 34 in round three) and due to the effects of a more sensitive test, whereas for older age groups (≥45 years old) the NNS decreases due to the use of a conditional 10‐year interval. This means that although the efficacy of the total programme is stable, the harms–benefits balance in younger age groups is increasing, which suggests an opportunity for optimisation of the screening programme.

### 4.2 Strengths and limitations

This study has several strengths. First, the study was performed with a well‐validated simulation model, using peer‐reviewed literature and Dutch population data for calibration and as direct inputs. The model has been used in many studies to give an insight in the consequences of policy changes on the harms and benefits of screening.^11^, ^25^, ^26^ Another strength of the study is that the most realistic assumptions were made for test characteristics and screen behaviour, based on the Dutch population. This makes the study results directly applicable for policy makers and healthcare providers. Finally, extensive sensitivity analyses were performed to eliminate possible changes in the results due to future uncertainties.

Our study also has some limitations. The screening behaviour was assumed to remain constant over time. However, this behaviour might be dynamic, especially during the first rounds of a new screening programme. We showed in a sensitivity analysis that attendance does not affect the presented outcomes. Another important limitation is that we did not take into account the effects of the Covid‐19 pandemic. Screening programmes in several countries, including the Netherlands, have been disrupted due to this pandemic.^27^ Although these disruptions have led to short‐term policy changes, such as wider use of the self‐test, this is not expected to have major effects on the harms and benefits of screening over the coming decades.^28^ Thirdly, because of the dynamic situation in the first few rounds of a new screening programme and the upcoming inflow of vaccinated women into the programme, it is likely that policy changes will be made further to optimise the harms and benefits. We did not take these changes into account in our study. Other modelling studies have been done to compare the balance between harms and benefits of potential policy changes.^26^, ^29^

### 4.3 Interpretation

To our knowledge, one other modelling study has been published with a similar objective and study design: Pesola et al. investigated the impact of HPV testing on colposcopy services and number of CIN2^+^ in Wales, starting from the first round of HPV vaccinated women.^30^ The authors found that the number of colposcopies rises in the first round of HPV testing in vaccinated cohorts and decreases by 30–40% in subsequent rounds. They found that CIN2^+^ diagnoses will decrease by 50–60% after the first round. Although we also found a decrease after the first screening round in both of these outcomes, the decrease in our results is not as substantial. This could be due to the fact that the Netherlands has a relatively low screening attendance and vaccination uptake compared with Wales. This leads to more colposcopy referrals and CIN2^+^ lesions in the subsequent HPV screening rounds. Comparison of the two studies clearly illustrates the effect of vaccination uptake and screening attendance on the changes in colposcopy referrals and CIN2^+^ lesions.

Veijalainen and colleagues (an observational population‐based study in Finland) found that the number of colposcopies performed decreased over the first two screening rounds from 4.0% to 2.9% of participating women.^31^ Although we found a decrease in the number of colposcopies over the rounds, we did not observe a decrease in colposcopies as a percentage of primary screens. Veijalainen and colleagues found, similar to our results, that the percentage of CIN2^+^ diagnoses was not significantly different between the two screening rounds. Bulkmans and colleagues found in the Dutch POBASCAM trial that the number of CIN3^+^ as a percentage of participants decreased significantly over the first two screening rounds.^32^ In our study we found a slight decrease in the number of CIN3^+^ lesions, but CIN3^+^ as a percentage of primary screens increased. Most of the differences between the current study and these publications can be explained by the fact that in the current study, fewer women are invited for colposcopy in the second and subsequent rounds, due to the 10‐year interval for women over 40 years old, which causes a decrease in primary tests over the first rounds.

## 5 CONCLUSION

We found that both harms and benefits of screening will decrease over the first three screening rounds. It is essential for stakeholders to take this into account for health services planning over the coming years. Importantly, the efficiency of the screening programme overall does not change substantially over the first and subsequent screening rounds. For younger women, however, the efficiency will decrease, which is compensated by an increased efficiency in older women due to longer screening intervals after age 40. This shows the importance of longer screening intervals for low‐risk women to maintain the same level of efficiency in the total programme. It will be necessary to keep monitoring the effects of the screening programme in the future, to ensure that the balance between harms and benefits remains favourable.

## CONFLICT OF INTERESTs

All authors report receiving funding from the Dutch National Institute for Public Health and the Environment for the conduct of this study. Completed disclosure of interest forms are available to view online as supporting information.

## AUTHOR CONTRIBUTIONS

SK wrote the first draft of the manuscript with contributions from EELJ and IMCMdK. SK and EELJ did the analyses. CAA coordinated the analysis of data for input into the model. All authors edited and approved the final version of the article. SK, EELJ, CAA and IMCMdK contributed to the development and conduct of the study. The corresponding author attests that all listed authors meet authorship criteria and that no others meeting the criteria have been omitted. SK and IMCMdK are the guarantors.

## ETHICAL APPROVAL

Ethical approval by a medical ethical committee was not required under Dutch law as no patients were involved in the development of the research and only non‐identifiable data were used for this study.

## Supporting information


Supplementary Material S1

ICMJE

ICMJE

ICMJE

ICMJE

## Data Availability

The data that support the findings of this study are available from the corresponding author upon reasonable request.
