# Supplementary material for: Shift in harms and benefits of cervical cancer screening in the era of HPV screening and vaccination: a modelling study
Source: BJOG. 2022 May 6;129(11):1862–9. doi: 10.1111/1471-0528.17190 (PMC9541905; doi:10.1111/1471-0528.17190)
Supplement: Supplementary file 1 — Supplementary Material S1 [file BJO-129-1862-s004.docx]

**APPENDICES**

*Supplement to: S Kaljouw, EEJ Jansen, CA Aitken, IMCM de Kok Shift in harms and benefits of cervical cancer screening in the era of HPV screening and vaccination: A modelling study*

[**Appendix S1: Model Assumptions 2**](#_Toc91165273)

[**Appendix S2: Base case results by age 11**](#_Toc91165274)

# Appendix S1: Model Assumptions

***Table S1: Transition probabilities (regression and progression) per HPV type, age group and current state, as defined in MISCAN-Cervix.***

| **HPV** | **Age*** | **Regression** | | **Probability** | **Progression** | | **Probability** |
| --- | --- | --- | --- | --- | --- | --- | --- |
| **type** |  | **From** | **To** |  | **From** | **To** |  |
| HPV 16 | 15 | HPV 16 | No HPV | 0.968 | HPV 16 | CIN 1 | 0.032 |
| HPV 16 | 25 | HPV 16 | No HPV | 0.978 | HPV 16 | CIN 1 | 0.022 |
| HPV 16 | 35 | HPV 16 | No HPV | 0.886 | HPV 16 | CIN 1 | 0.114 |
| HPV 16 | 50 | HPV 16 | No HPV | 0.762 | HPV 16 | CIN 1 | 0.238 |
| HPV 16 | 75 | HPV 16 | No HPV | 0.993 | HPV 16 | CIN 1 | 0.007 |
| HPV 18 | 15 | HPV 18 | No HPV | 0.943 | HPV 18 | CIN 1 | 0.057 |
| HPV 18 | 25 | HPV 18 | No HPV | 0.962 | HPV 18 | CIN 1 | 0.038 |
| HPV 18 | 35 | HPV 18 | No HPV | 0.801 | HPV 18 | CIN 1 | 0.199 |
| HPV 18 | 50 | HPV 18 | No HPV | 0.582 | HPV 18 | CIN 1 | 0.418 |
| HPV 18 | 75 | HPV 18 | No HPV | 0.988 | HPV 18 | CIN 1 | 0.012 |
| HPV 9V | 15 | HPV 9V | No HPV | 0.975 | HPV 9V | CIN 1 | 0.025 |
| HPV 9V | 25 | HPV 9V | No HPV | 0.983 | HPV 9V | CIN 1 | 0.017 |
| HPV 9V | 35 | HPV 9V | No HPV | 0.913 | HPV 9V | CIN 1 | 0.087 |
| HPV 9V | 50 | HPV 9V | No HPV | 0.817 | HPV 9V | CIN 1 | 0.183 |
| HPV 9V | 75 | HPV 9V | No HPV | 0.995 | HPV 9V | CIN 1 | 0.005 |
| HPVOHR | 15 | HPVOHR | No HPV | 0.975 | HPVOHR | CIN 1 | 0.025 |
| HPVOHR | 25 | HPVOHR | No HPV | 0.983 | HPVOHR | CIN 1 | 0.017 |
| HPVOHR | 35 | HPVOHR | No HPV | 0.913 | HPVOHR | CIN 1 | 0.087 |
| HPVOHR | 50 | HPVOHR | No HPV | 0.818 | HPVOHR | CIN 1 | 0.182 |
| HPVOHR | 75 | HPVOHR | No HPV | 0.995 | HPVOHR | CIN 1 | 0.005 |
| HPV 16 | 20 | CIN 1 | HPV 16 | 0.556 | CIN 1 | CIN 2 | 0.444 |
| HPV 16 | 35 | CIN 1 | HPV 16 | 0.038 | CIN 1 | CIN 2 | 0.962 |
| HPV 16 | 50 | CIN 1 | HPV 16 | 0.519 | CIN 1 | CIN 2 | 0.481 |
| HPV 16 | 65 | CIN 1 | HPV 16 | 0.869 | CIN 1 | CIN 2 | 0.131 |
| HPV 18 | 20 | CIN 1 | HPV 18 | 0.880 | CIN 1 | CIN 2 | 0.120 |
| HPV 18 | 35 | CIN 1 | HPV 18 | 0.741 | CIN 1 | CIN 2 | 0.259 |
| HPV 18 | 50 | CIN 1 | HPV 18 | 0.870 | CIN 1 | CIN 2 | 0.130 |
| HPV 18 | 65 | CIN 1 | HPV 18 | 0.965 | CIN 1 | CIN 2 | 0.035 |
| HPV 9V | 20 | CIN 1 | HPV 9V | 0.736 | CIN 1 | CIN 2 | 0.264 |
| HPV 9V | 35 | CIN 1 | HPV 9V | 0.427 | CIN 1 | CIN 2 | 0.573 |
| HPV 9V | 50 | CIN 1 | HPV 9V | 0.714 | CIN 1 | CIN 2 | 0.286 |
| HPV 9V | 65 | CIN 1 | HPV 9V | 0.922 | CIN 1 | CIN 2 | 0.078 |
| HPVOHR | 20 | CIN 1 | HPVOHR | 0.877 | CIN 1 | CIN 2 | 0.123 |
| HPVOHR | 35 | CIN 1 | HPVOHR | 0.732 | CIN 1 | CIN 2 | 0.268 |
| HPVOHR | 50 | CIN 1 | HPVOHR | 0.866 | CIN 1 | CIN 2 | 0.134 |
| HPVOHR | 65 | CIN 1 | HPVOHR | 0.964 | CIN 1 | CIN 2 | 0.036 |
| NoHPV | 20 | CIN 1 | No HPV | 0.762 | CIN 1 | CIN 2 | 0.238 |
| NoHPV | 35 | CIN 1 | No HPV | 0.485 | CIN 1 | CIN 2 | 0.515 |
| NoHPV | 50 | CIN 1 | No HPV | 0.743 | CIN 1 | CIN 2 | 0.257 |
| NoHPV | 65 | CIN 1 | No HPV | 0.930 | CIN 1 | CIN 2 | 0.070 |
| HPV 16 | 20 | CIN 2 | CIN 1 | 0.518 | CIN 2 | CIN 3 | 0.482 |
| HPV 16 | 35 | CIN 2 | CIN 1 | 0.459 | CIN 2 | CIN 3 | 0.541 |
| HPV 16 | 50 | CIN 2 | CIN 1 | 0.766 | CIN 2 | CIN 3 | 0.234 |
| HPV 16 | 65 | CIN 2 | CIN 1 | 0.704 | CIN 2 | CIN 3 | 0.296 |
| HPV 18 | 20 | CIN 2 | CIN 1 | 0.815 | CIN 2 | CIN 3 | 0.185 |
| HPV 18 | 35 | CIN 2 | CIN 1 | 0.792 | CIN 2 | CIN 3 | 0.208 |
| HPV 18 | 50 | CIN 2 | CIN 1 | 0.910 | CIN 2 | CIN 3 | 0.090 |
| HPV 18 | 65 | CIN 2 | CIN 1 | 0.886 | CIN 2 | CIN 3 | 0.114 |
| HPV 9V | 20 | CIN 2 | CIN 1 | 0.657 | CIN 2 | CIN 3 | 0.343 |
| HPV 9V | 35 | CIN 2 | CIN 1 | 0.615 | CIN 2 | CIN 3 | 0.385 |
| HPV 9V | 50 | CIN 2 | CIN 1 | 0.833 | CIN 2 | CIN 3 | 0.167 |
| HPV 9V | 65 | CIN 2 | CIN 1 | 0.789 | CIN 2 | CIN 3 | 0.211 |
| HPVOHR | 20 | CIN 2 | CIN 1 | 0.729 | CIN 2 | CIN 3 | 0.271 |
| HPVOHR | 35 | CIN 2 | CIN 1 | 0.696 | CIN 2 | CIN 3 | 0.304 |
| HPVOHR | 50 | CIN 2 | CIN 1 | 0.868 | CIN 2 | CIN 3 | 0.132 |
| HPVOHR | 65 | CIN 2 | CIN 1 | 0.833 | CIN 2 | CIN 3 | 0.167 |
| NoHPV | 20 | CIN 2 | CIN 1 | 0.609 | CIN 2 | CIN 3 | 0.391 |
| NoHPV | 35 | CIN 2 | CIN 1 | 0.561 | CIN 2 | CIN 3 | 0.439 |
| NoHPV | 50 | CIN 2 | CIN 1 | 0.810 | CIN 2 | CIN 3 | 0.190 |
| NoHPV | 65 | CIN 2 | CIN 1 | 0.760 | CIN 2 | CIN 3 | 0.240 |
| HPV 16 | 20 | CIN 3 | CIN 2 | 0.930 | CIN 3 | CC | 0.070 |
| HPV 16 | 35 | CIN 3 | CIN 2 | 0.882 | CIN 3 | CC | 0.118 |
| HPV 16 | 50 | CIN 3 | CIN 2 | 0.865 | CIN 3 | CC | 0.135 |
| HPV 16 | 65 | CIN 3 | CIN 2 | 0.090 | CIN 3 | CC | 0.910 |
| HPV 18 | 20 | CIN 3 | CIN 2 | 0.561 | CIN 3 | CC | 0.439 |
| HPV 18 | 35 | CIN 3 | CIN 2 | 0.254 | CIN 3 | CC | 0.746 |
| HPV 18 | 50 | CIN 3 | CIN 2 | 0.147 | CIN 3 | CC | 0.853 |
| HPV 18 | 65 | CIN 3 | CIN 2 | 0.090 | CIN 3 | CC | 0.910 |
| HPV 9V | 20 | CIN 3 | CIN 2 | 0.970 | CIN 3 | CC | 0.030 |
| HPV 9V | 35 | CIN 3 | CIN 2 | 0.949 | CIN 3 | CC | 0.051 |
| HPV 9V | 50 | CIN 3 | CIN 2 | 0.942 | CIN 3 | CC | 0.058 |
| HPV 9V | 65 | CIN 3 | CIN 2 | 0.090 | CIN 3 | CC | 0.910 |
| HPVOHR | 20 | CIN 3 | CIN 2 | 0.981 | CIN 3 | CC | 0.019 |
| HPVOHR | 35 | CIN 3 | CIN 2 | 0.968 | CIN 3 | CC | 0.032 |
| HPVOHR | 50 | CIN 3 | CIN 2 | 0.963 | CIN 3 | CC | 0.037 |
| HPVOHR | 65 | CIN 3 | CIN 2 | 0.090 | CIN 3 | CC | 0.910 |
| No HPV | 20 | CIN 3 | CIN 2 | 1.000 | CIN 3 | CC | 0.000** |
| No HPV | 35 | CIN 3 | CIN 2 | 1.000 | CIN 3 | CC | 0.000** |
| No HPV | 50 | CIN 3 | CIN 2 | 1.000 | CIN 3 | CC | 0.000** |
| No HPV | 65 | CIN 3 | CIN 2 | 1.000 | CIN 3 | CC | 0.000** |

*hrHPV = high-risk human papillomavirus; CIN = cervical intraepithelial neoplasia*; *CC =* *cervical cancer; HPV 9V = HPV-31/33/45/52/58; HPVOHR = HPV-35/39/51/56/59/66/68*

* Remaining ages are linearly interpolated based on the values in the table.
** CIN 3 lesions can never transition to cervical cancer without an HPV infection

***Table S2: cytology/HPV test characteristics per stage.***

|  |  | **Probability of a positive test result** | | |
| --- | --- | --- | --- | --- |
| **HPV infection present** | **Disease status** | *Cytology ≥ASC-US** | *Cytology ≥HSIL** | *Positive hrHPV-test*** |
| ≥1 HPV infection | no CIN present | 17.1% | 0.0% | 55.0% |
| ≥1 HPV infection | CIN1 | 36.2% | 2.6% | 72.0% |
| ≥1 HPV infection | CIN2 | 37.1% | 10.7% | 94.0% |
| ≥1 HPV infection | CIN3 | 75.4% | 51.6% | 94.0% |
| ≥1 HPV infection | FIGO 1A | 85.1% | 64.7% | 94.0% |
| ≥1 HPV infection | FIGO 1B | 85.1% | 64.7% | 94.0% |
| ≥1 HPV infection | FIGO 2 | 85.1% | 64.7% | 94.0% |
| ≥1 HPV infection | FIGO 3 | 85.1% | 64.7% | 94.0% |
| ≥1 HPV infection | FIGO 4 | 85.1% | 64.7% | 94.0% |
| No HPV | no CIN present | 0.6% | 0.04% | 0.0% |
| No HPV | CIN1 | 36.2% | 2.6% | 0.0% |
| No HPV | CIN2 | 37.1% | 10.7% | 0.0% |
| No HPV | CIN3 | 75.4% | 51.6% | 0.0% |
| No HPV | FIGO 1A | 85.1% | 64.7% | 0.0% |
| No HPV | FIGO 1B | 85.1% | 64.7% | 0.0% |
| No HPV | FIGO 2 | 85.1% | 64.7% | 0.0% |
| No HPV | FIGO 3 | 85.1% | 64.7% | 0.0% |
| No HPV | FIGO 4 | 85.1% | 64.7% | 0.0% |

* Probability to test positive the first time a women with this lesion present attends screening. 12% of the CIN lesions will be missed systematically over time.
** The same test characteristics are assumed for GP smears as for self-sampling kits

hrHPV = high-risk human papillomavirus; CIN = cervical intraepithelial neoplasia; ASC-US = Atypical squamous cells of undetermined significance; HSIL = High-grade squamous intraepithelial lesion, FIGO = International Federation of Gynecology and Obstetrics

***Table S3: Assumptions screen behaviour for base case analysis and sensitivity analysis.***

| **Screen behaviour** | **Current programme** | **Sensitivity analysis (higher attendance)** |
| --- | --- | --- |
| Attendance to office-based test by age*** |  |  |
| 30 | 41.7% | 52.3% |
| 35 | 47.3% | 57.9% |
| 40 | 54.5% | 64.3% |
| 45 | 57.2%*/13.3%** | 67.6% |
| 50 | 58.6% | 70.4% |
| 55 | 58.6%*/9.5%** | 69.6% |
| 60 | 58.0% | 66.8% |
| 65 | NA*/1.4%** | NA |
| Self-test attendance by age *** |  |  |
|  |  |  |
| 30 | 4.5% | NA |
| 35 | 3.7% | NA |
| 40 | 3.5% | NA |
| 45 | 3.4%*/0.5%** | NA |
| 50 | 3.5% | NA |
| 55 | 4.2%*/0.9%** | NA |
| 60 | 4.4% | NA |
| 65 | NA*/0.1%** | NA |
| Adherence  primary triage cytology after positive self-test | 90.0% | NA |
| Adherence secondary triage cytology |  |  |
| - 6 months after primary office-based test | 86.7% | 92.2% |
| - 6 months after primary self-test | 82.8% | NA |
| Adherence for referral to colposcopy after |  |  |
| - direct referral (ASC-US/LSIL) | 88.4% | NA |
| - direct referral (HSIL+) | 96.9% | 97.0% |
| - referral 6 months after primary test (ASC-US/LSIL) | 88.4% | 97.5% |
| - referral 6 months after primary test (HSIL+) | 96.9% | 97.5% |

* Attendance rate (percentage of women in age group) in the first screening round (2017-2021)

** Attendance rate (percentage of women in age group) from the second screening round (from 2022).

*** Simulated attendance rate in all women within age group with an exception of women who have had a hysterectomy and women who have been diagnosed with cervical cancer.

HPV = human papillomavirus; NA = not applicable

***Table S4: Vaccination rates (fully vaccinated, 2 or 3 doses)***

|  | **Calendar year** | | | | | | | | | | |
| --- | --- | --- | --- | --- | --- | --- | --- | --- | --- | --- | --- |
| **Birth cohort** | 2008 | 2009 | 2010 | 2011 | 2012 | 2013 | 2014 | 2015 | 2016 | 2017 | 2018 |
| 1993 | 0 | 0 | 0.49 | 0 | 0 | 0 | 0 | 0 | 0 | 0 | 0 |
| 1994 | 0 | 0 | 0.525 | 0 | 0 | 0 | 0 | 0 | 0 | 0 | 0 |
| 1995 | 0 | 0 | 0.538 | 0 | 0 | 0 | 0 | 0 | 0 | 0 | 0 |
| 1996 | 0 | 0 | 0.542 | 0 | 0 | 0 | 0 | 0 | 0 | 0 | 0 |
| 1997 | 0 | 0 | 0 | 0.56 | 0 | 0 | 0 | 0 | 0 | 0 | 0 |
| 1998 | 0 | 0 | 0 | 0 | 0.581 | 0 | 0 | 0 | 0 | 0 | 0 |
| 1999 | 0 | 0 | 0 | 0 | 0 | 0.589 | 0 | 0 | 0 | 0 | 0 |
| 2000 | 0 | 0 | 0 | 0 | 0 | 0 | 0.61 | 0 | 0 | 0 | 0 |
| 2001 | 0 | 0 | 0 | 0 | 0 | 0 | 0 | 0.61 | 0 | 0 | 0 |
| 2002 | 0 | 0 | 0 | 0 | 0 | 0 | 0 | 0 | 0.534 | 0 | 0 |
| 2003 | 0 | 0 | 0 | 0 | 0 | 0 | 0 | 0 | 0 | 0.455 | 0 |
| >=2004 | 0 | 0 | 0 | 0 | 0 | 0 | 0 | 0 | 0 | 0 | 0.455* |

* Women are vaccinated at age 13.

# Appendix S2: Base case results by age

***Table S1a: Base case results for capacity, harms and benefits by age group.****The results in this table clearly illustrate the effect of vaccination on CIN2+ detection in the youngest age groups: 30-34 (2^nd^ and 3^rd^ round) and 35-39 (3^rd^ round).*

| **First screening round** | **30-34** | **35-39** | **40-44** | **45-49** | **50-54** | **55-59** | **60-64** | **65-69** |
| --- | --- | --- | --- | --- | --- | --- | --- | --- |
| hrHPV GP tests | 231,655 | 249,495 | 284,114 | 325,554 | 369,279 | 360,407 | 312,743 | 0 |
| hrHPV self-tests | 24,868 | 19,414 | 18,114 | 19,260 | 21,907 | 25,595 | 23,582 | 0 |
| hrHPV positive | 71,879 | 38,728 | 32,653 | 30,321 | 25,760 | 21,961 | 17,154 | 0 |
| Cytology | 116,001 | 60,940 | 51,119 | 48,302 | 40,961 | 35,373 | 27,842 | 0 |
| Direct Referral | 15,350 | 10,795 | 9,613 | 8,818 | 7,411 | 5,751 | 4,115 | 0 |
| Indirect referral | 6,606 | 3,913 | 3,349 | 3,355 | 2,887 | 2,389 | 1,732 | 0 |
| No CIN | 12,682 | 5,240 | 4,146 | 3,758 | 3,107 | 2,953 | 2,538 | 0 |
| CIN1 | 3,916 | 3,057 | 2,811 | 3,263 | 3,260 | 2,758 | 1,887 | 0 |
| CIN2 | 2,404 | 2,827 | 2,439 | 2,322 | 1,943 | 1,284 | 663 | 0 |
| CIN3 | 2,668 | 3,363 | 3,307 | 2,581 | 1,773 | 974 | 592 | 0 |
| CC screen detected | 100 | 149 | 207 | 192 | 175 | 138 | 139 | 0 |
| CC clinically detected | 137 | 210 | 270 | 303 | 277 | 240 | 202 | 238 |
| Hysterectomy | 175 | 196 | 185 | 164 | 130 | 86 | 52 | 0 |
| Large excisions | 5,049 | 5,647 | 5,256 | 4,720 | 3,800 | 2,536 | 1,527 | 0 |
| Biopsies | 3,665 | 3,316 | 3,037 | 3,197 | 2,966 | 2,329 | 1,520 | 0 |
| Treatment related pain | 3,383 | 3,783 | 3,522 | 3,162 | 2,546 | 1,699 | 1,023 | 0 |
| Treatment related bleeding | 3,888 | 4,348 | 4,047 | 3,634 | 2,926 | 1,953 | 1,176 | 0 |
| Treatment related discharge | 3,181 | 3,557 | 3,311 | 2,974 | 2,394 | 1,598 | 962 | 0 |
| **Second screening round** | **30-34** | **35-39** | **40-44** | **45-49** | **50-54** | **55-59** | **60-64** | **65-69** |
| hrHPV GP tests | 227,127 | 260,989 | 283,527 | 70,283 | 322,347 | 62,880 | 346,747 | 8,482 |
| hrHPV self-tests | 24,393 | 20,323 | 18,116 | 3,277 | 19,126 | 6,239 | 26,075 | 857 |
| hrHPV positive | 63,045 | 39,458 | 31,628 | 7,995 | 22,329 | 5,038 | 18,893 | 884 |
| Cytology | 104,572 | 62,681 | 50,104 | 13,395 | 35,593 | 8,888 | 30,579 | 1,327 |
| Direct Referral | 13,074 | 10,589 | 8,913 | 2,665 | 6,339 | 1,502 | 4,497 | 254 |
| Indirect referral | 5,861 | 3,741 | 3,038 | 929 | 2,323 | 623 | 1,737 | 61 |
| No CIN | 11,766 | 5,491 | 4,137 | 870 | 2,622 | 576 | 2,751 | 69 |
| CIN1 | 3,258 | 3,089 | 2,722 | 800 | 2,859 | 618 | 2,067 | 74 |
| CIN2 | 1,900 | 2,723 | 2,267 | 706 | 1,606 | 388 | 673 | 49 |
| CIN3 | 1,945 | 2,896 | 2,665 | 1,109 | 1,429 | 457 | 603 | 78 |
| CC screen detected | 66 | 132 | 161 | 109 | 146 | 85 | 140 | 45 |
| CC clinically detected | 109 | 228 | 282 | 280 | 271 | 252 | 230 | 250 |
| Hysterectomy | 135 | 180 | 160 | 59 | 108 | 29 | 55 | 4 |
| Large excisions | 3,907 | 5,208 | 4,587 | 1,639 | 3,162 | 828 | 1,601 | 121 |
| Biopsies | 2,980 | 3,235 | 2,832 | 895 | 2,555 | 589 | 1,641 | 73 |
| Treatment related pain | 2,618 | 3,490 | 3,074 | 1,098 | 2,119 | 555 | 1,073 | 81 |
| Treatment related bleeding | 3,008 | 4,010 | 3,532 | 1,262 | 2,435 | 638 | 1,233 | 93 |
| Treatment related discharge | 2,461 | 3,281 | 2,890 | 1,032 | 1,992 | 522 | 1,009 | 76 |
| **Third screening round** | **30-34** | **35-39** | **40-44** | **45-49** | **50-54** | **55-59** | **60-64** | **65-69** |
| hrHPV GP tests | 215,781 | 255,897 | 296,344 | 70,238 | 288,019 | 54,981 | 345,193 | 9,390 |
| hrHPV self-tests | 23,192 | 19,938 | 18,941 | 3,294 | 17,092 | 5,443 | 26,028 | 937 |
| hrHPV positive | 56,622 | 33,815 | 32,799 | 7,990 | 20,616 | 4,410 | 19,165 | 951 |
| Cytology | 93,171 | 54,697 | 51,755 | 12,238 | 32,207 | 6,970 | 30,793 | 1,484 |
| Direct Referral | 11,519 | 8,744 | 9,266 | 2,693 | 6,239 | 1,290 | 4,794 | 283 |
| Indirect referral | 5,090 | 3,270 | 3,115 | 723 | 2,041 | 418 | 1,762 | 72 |
| No CIN | 10,596 | 4,943 | 4,294 | 765 | 2,342 | 449 | 2,716 | 81 |
| CIN1 | 2,831 | 2,608 | 2,782 | 723 | 2,514 | 492 | 2,095 | 84 |
| CIN2 | 1,557 | 2,177 | 2,335 | 680 | 1,516 | 324 | 725 | 55 |
| CIN3 | 1,578 | 2,205 | 2,795 | 1,132 | 1,727 | 370 | 829 | 83 |
| CC screen detected | 47 | 82 | 174 | 115 | 180 | 74 | 191 | 52 |
| CC clinically detected | 74 | 195 | 303 | 302 | 241 | 255 | 253 | 269 |
| Hysterectomy | 112 | 141 | 166 | 58 | 113 | 24 | 64 | 5 |
| Large excisions | 3,243 | 4,112 | 4,760 | 1,621 | 3,240 | 679 | 1,831 | 132 |
| Biopsies | 2,543 | 2,665 | 2,909 | 835 | 2,341 | 470 | 1,707 | 83 |
| Treatment related pain | 2,172 | 2,755 | 3,189 | 1,086 | 2,171 | 455 | 1,227 | 89 |
| Treatment related bleeding | 2,497 | 3,167 | 3,665 | 1,248 | 2,495 | 523 | 1,410 | 102 |
| Treatment related discharge | 2,043 | 2,591 | 2,999 | 1,021 | 2,041 | 428 | 1,153 | 83 |

hrHPV = high-risk human papillomavirus; CIN = cervical intraepithelial neoplasia; CC = cervical cancer; GP = general practitioner.

***Table S1b: Base case results for difference in capacity, harms and benefits by age group with respect to the first screening round.****The results in this table clearly illustrate the effect of vaccination on CIN2+ detection in the youngest age groups: 30-34 (2^nd^ and 3^rd^ round) and 35-39 (3^rd^ round).*

| **Second screening round** | **30-34** | **35-39** | **40-44** | **45-49** | **50-54** | **55-59** | **60-64** |
| --- | --- | --- | --- | --- | --- | --- | --- |
| hrHPV GP tests | -2.0% | +4.6% | -0.2% | -78.4% | -12.7% | -82.6% | +10.9% |
| hrHPV self-tests | -1.9% | +4.7% | +0.0% | -83.0% | -12.7% | -75.6% | +10.6% |
| hrHPV positive | -12.3% | +1.9% | -3.1% | -73.6% | -13.3% | -77.1% | +10.1% |
| Cytology | -9.9% | +2.9% | -2.0% | -72.3% | -13.1% | -74.9% | +9.8% |
| Direct Referral | -14.8% | -1.9% | -7.3% | -69.8% | -14.5% | -73.9% | +9.3% |
| Indirect referral | -11.3% | -4.4% | -9.3% | -72.3% | -19.5% | -73.9% | +0.3% |
| No CIN | -7.2% | +4.8% | -0.2% | -76.8% | -15.6% | -80.5% | +8.4% |
| CIN1 | -16.8% | +1.1% | -3.2% | -75.5% | -12.3% | -77.6% | +9.6% |
| CIN2 | -20.9% | -3.7% | -7.1% | -69.6% | -17.3% | -69.8% | +1.4% |
| CIN3 | -27.1% | -13.9% | -19.4% | -57.0% | -19.4% | -53.1% | +1.9% |
| CC screen detected | -33.5% | -11.4% | -22.2% | -43.2% | -16.6% | -38.1% | +0.3% |
| CC clinically detected | -20.6% | +8.5% | +4.7% | -7.6% | -2.2% | +4.8% | +13.6% |
| CIN2+ lesions | -24.3% | -8.7% | -13.6% | -59.2% | -17.2% | -55.2% | +3.0% |
| Hysterectomy | -22.9% | -8.5% | -13.6% | -64.2% | -16.7% | -66.3% | +5.1% |
| Large excisions | -22.6% | -7.8% | -12.7% | -65.3% | -16.8% | -67.3% | +4.8% |
| Biopsies | -18.7% | -2.5% | -6.7% | -72.0% | -13.9% | -74.7% | +7.9% |
| Treatment related pain | -22.6% | -7.8% | -12.7% | -65.3% | -16.8% | -67.3% | +4.8% |
| Treatment related bleeding | -22.6% | -7.8% | -12.7% | -65.3% | -16.8% | -67.3% | +4.8% |
| Treatment related discharge | -22.6% | -7.8% | -12.7% | -65.3% | -16.8% | -67.3% | +4.8% |
| **Third screening round** | **30-34** | **35-39** | **40-44** | **45-49** | **50-54** | **55-59** | **60-64** |
| hrHPV GP tests | -6.9% | +2.6% | +4.3% | -78.4% | -22.0% | -84.7% | +10.4% |
| hrHPV self-tests | -6.7% | +2.7% | +4.6% | -82.9% | -22.0% | -78.7% | +10.4% |
| hrHPV positive | -21.2% | -12.7% | +0.4% | -73.6% | -20.0% | -79.9% | +11.7% |
| Cytology | -19.7% | -10.2% | +1.2% | -74.7% | -21.4% | -80.3% | +10.6% |
| Direct Referral | -25.0% | -19.0% | -3.6% | -69.5% | -15.8% | -77.6% | +16.5% |
| Indirect referral | -22.9% | -16.4% | -7.0% | -78.5% | -29.3% | -82.5% | +1.7% |
| No CIN | -16.4% | -5.7% | +3.6% | -79.6% | -24.6% | -84.8% | +7.0% |
| CIN1 | -27.7% | -14.7% | -1.0% | -77.8% | -22.9% | -82.2% | +11.0% |
| CIN2 | -35.2% | -23.0% | -4.3% | -70.7% | -22.0% | -74.8% | +9.3% |
| CIN3 | -40.8% | -34.4% | -15.5% | -56.1% | -2.6% | -62.0% | +40.1% |
| CC screen detected | -53.3% | -44.8% | -15.9% | -40.0% | +2.9% | -46.7% | +37.1% |
| CC clinically detected | -46.3% | -6.9% | +12.6% | -0.4% | -13.0% | +6.5% | +25.0% |
| CIN2+ lesions | -38.7% | -28.9% | -9.9% | -58.7% | -12.1% | -61.2% | +25.1% |
| Hysterectomy | -36.0% | -28.0% | -10.2% | -64.4% | -13.0% | -72.4% | +22.0% |
| Large excisions | -35.8% | -27.2% | -9.4% | -65.7% | -14.7% | -73.2% | +19.9% |
| Biopsies | -30.6% | -19.6% | -4.2% | -73.9% | -21.1% | -79.8% | +12.3% |
| Treatment related pain | -35.8% | -27.2% | -9.4% | -65.7% | -14.7% | -73.2% | +19.9% |
| Treatment related bleeding | -35.8% | -27.2% | -9.4% | -65.7% | -14.7% | -73.2% | +19.9% |
| Treatment related discharge | -35.8% | -27.2% | -9.4% | -65.7% | -14.7% | -73.2% | +19.9% |

GP: general practitioner, hrHPV: high-risk Human Papillomavirus, CIN: Cervical Intraepithelial Neoplasia, CC: cervical cancer

***Table S2a: Base case results for efficiency by age group.****The results in this table clearly illustrate the effect of vaccination on efficiency in the youngest age groups: 30-34 (2^nd^ and 3^rd^ round) and 35-39 (3^rd^ round). It also shows the effect of the conditional ten-year interval for older women.*

| **First screening round** | **30-34** | **35-39** | **40-44** | **45-49** | **50-54** | **55-59** | **60-64** | **65-69** |
| --- | --- | --- | --- | --- | --- | --- | --- | --- |
| Diagnoses per hrHPV test | 0.020 | 0.024 | 0.020 | 0.015 | 0.010 | 0.006 | 0.004 | N/A |
| Diagnoses per hrHPV positive | 0.072 | 0.164 | 0.182 | 0.168 | 0.151 | 0.109 | 0.081 | N/A |
| Diagnoses per cytology test | 0.045 | 0.104 | 0.116 | 0.105 | 0.095 | 0.068 | 0.050 | N/A |
| Diagnoses per referral | 0.236 | 0.431 | 0.459 | 0.419 | 0.378 | 0.294 | 0.238 | N/A |
| NNS CIN2+ | 50 | 42 | 51 | 68 | 101 | 161 | 241 | N/A |
| NNS CIN3+ | 93 | 77 | 86 | 124 | 201 | 347 | 460 | N/A |
| **Second screening round** | **30-34** | **35-39** | **40-44** | **45-49** | **50-54** | **55-59** | **60-64** | **65-69** |
| Diagnoses per hrHPV test | 0.016 | 0.020 | 0.017 | 0.026 | 0.009 | 0.013 | 0.004 | 0.018 |
| Diagnoses per hrHPV positive | 0.062 | 0.146 | 0.161 | 0.241 | 0.142 | 0.185 | 0.075 | 0.194 |
| Diagnoses per cytology test | 0.037 | 0.092 | 0.102 | 0.144 | 0.089 | 0.105 | 0.046 | 0.130 |
| Diagnoses per referral | 0.207 | 0.401 | 0.426 | 0.535 | 0.367 | 0.438 | 0.227 | 0.546 |
| NNS CIN2+ | 64 | 49 | 59 | 38 | 107 | 74 | 263 | 54 |
| NNS CIN3+ | 125 | 93 | 107 | 60 | 217 | 127 | 502 | 76 |
| **Third screening round** | **30-34** | **35-39** | **40-44** | **45-49** | **50-54** | **55-59** | **60-64** | **65-69** |
| Diagnoses per hrHPV test | 0.013 | 0.016 | 0.017 | 0.026 | 0.011 | 0.013 | 0.005 | 0.018 |
| Diagnoses per hrHPV positive | 0.056 | 0.132 | 0.162 | 0.241 | 0.166 | 0.174 | 0.091 | 0.199 |
| Diagnoses per cytology test | 0.034 | 0.082 | 0.102 | 0.158 | 0.106 | 0.110 | 0.057 | 0.127 |
| Diagnoses per referral | 0.192 | 0.372 | 0.428 | 0.564 | 0.413 | 0.449 | 0.266 | 0.534 |
| NNS CIN2+ | 75 | 62 | 59 | 38 | 89 | 79 | 213 | 55 |
| NNS CIN3+ | 147 | 121 | 106 | 59 | 160 | 136 | 364 | 77 |

* PPV: positive predictive value of a referral
hrHPV: high-risk Human Papillomavirus, CIN: Cervical Intraepithelial Neoplasia, NNS: Number of women needed to screen to detect one CIN2+/3+ lesion

***Table S2b: Base case results for difference in efficiency by age group with respect to the first screening round.****The results in this table clearly illustrate the effect of vaccination on efficiency in the youngest age groups: 30-34 (2^nd^ and 3^rd^ round) and 35-39 (3^rd^ round). It also shows the effect of the conditional ten-year interval for older women.*

| **Second screening round** | **30-34** | **35-39** | **40-44** | **45-49** | **50-54** | **55-59** | **60-64** |
| --- | --- | --- | --- | --- | --- | --- | --- |
| Diagnoses per hrHPV test | -22.9% | -13.3% | -14.3% | +77.0% | -6.4% | +116.8% | -8.4% |
| Diagnoses per hrHPV positive | -13.8% | -11.0% | -11.7% | +43.2% | -5.7% | +69.3% | -7.8% |
| Diagnoses per cytology test | -16.1% | -11.8% | -12.7% | +36.2% | -5.9% | +54.5% | -7.6% |
| Diagnoses per referral | -12.3% | -6.9% | -7.2% | +27.9% | -2.8% | +48.8% | -4.8% |
| NNS CIN2+ | +29.7% | +15.3% | +16.7% | -43.5% | +6.8% | -53.9% | +9.2% |
| NNS CIN3+ | +35.0% | +21.3% | +24.1% | -51.4% | +8.0% | -63.3% | +9.1% |
| **Third screening round** | **30-34** | **35-39** | **40-44** | **45-49** | **50-54** | **55-59** | **60-64** |
| Diagnoses per hrHPV test | -33.9% | -31.4% | -14.6% | +77.4% | +12.8% | +104.5% | +13.4% |
| Diagnoses per hrHPV positive | -21.9% | -19.3% | -11.3% | +43.6% | +9.9% | +59.5% | +12.0% |
| Diagnoses per cytology test | -23.4% | -21.5% | -12.0% | +49.3% | +11.9% | +62.5% | +13.2% |
| Diagnoses per referral | -18.7% | -13.8% | -6.7% | +34.8% | +9.4% | +52.6% | +11.6% |
| NNS CIN2+ | +51.4% | +45.7% | +17.1% | -43.6% | -11.3% | -51.1% | -11.8% |
| NNS CIN3+ | +58.7% | +57.5% | +23.5% | -52.6% | -20.3% | -60.7% | -20.9% |

* PPV: positive predictive value of a referral
hrHPV: high-risk Human Papillomavirus, CIN: Cervical Intraepithelial Neoplasia, NNS: Number of women needed to screen to detect one CIN2+/3+ lesion
